# Antibiofilm Properties and Demineralization Suppression in Early Enamel Lesions Using Dental Coating Materials

**DOI:** 10.3390/antibiotics13010106

**Published:** 2024-01-22

**Authors:** Niraya Kornsombut, Shoji Takenaka, Maki Sotozono, Ryoko Nagata, Takako Ida, Jutharat Manuschai, Rui Saito, Ryouhei Takahashi, Yuichiro Noiri

**Affiliations:** 1 Department of Cariology, Operative Dentistry and Endodontics, Faculty of Dentistry, Graduate School of Medical and Dental Sciences, Niigata University, Niigata 951-8514, Japan; nirayak@nu.ac.th (N.K.); noiri@dent.niigata-u.ac.jp (Y.N.); 2 Department of Restorative Dentistry, Faculty of Dentistry, Naresuan University, Phitsanulok 65000, Thailand

**Keywords:** dental biofilm, dental coating material, ion incorporation, early enamel lesion, *Streptococcus mutans*

## Abstract

This study aimed to investigate the effects of dental coating materials on *Streptococcus mutans* biofilm formation. The test materials were PRG Barrier Coat (PRG), BioCoat Ca (BioC), and FluorDental Jelly (FluorJ). Bovine enamel specimens were demineralized to mimic early enamel lesions. The biofilm was developed on a specimen treated with one of the materials by using a modified Robbins device flow-cell system. Scanning electron and fluorescence confocal laser scanning microscopy, viable and total cell counts, and gene expression assessments of the antibiofilm were performed. Ion incorporation was analyzed using a wavelength-dispersive X-ray spectroscopy electron probe microanalyzer. All materials allowed biofilm formation but reduced its volume. FluorJ was the only material that inhibited biofilm accumulation and had a bactericidal effect, revealing 0.66 log CFU in viable cells and 1.23 log copy reduction in total cells compared with the untreated group after 24 h of incubation. The ions released from PRG varied depending on the element. BioC contributed to enamel remineralization by supplying calcium ions while blocking the acid produced from the biofilm. In summary, the dental coating materials physically prevented acid attacks from the biofilm while providing ions to the enamel to improve its mechanical properties.

## 1. Introduction

Anticariogenic properties refer to reducing the activity of oral bacteria to prevent the occurrence of dental caries. Remineralization strategies aim to increase the concentration of calcium (Ca) and phosphate (P) ions delivered to carious lesions or increase their concentrations in the plaque and saliva [1]. The increased concentrations of Ca and P ions help to repair and strengthen the enamel, which is the outer layer of the tooth that protects the tooth from decay. Fluoride is the gold standard when investigating tooth remineralization because it effectively prevents dental caries and promotes tooth remineralization [2]. The formation of dense crystalline Ca and P structures through mineral–fluoride interactions can prevent demineralization. In the early stages of caries formation, remineralization with fluoride treatment accelerates the synthesis of healthy minerals inside the enamel and inhibits the advancement of dental caries. However, the lack of fluoride retention on the enamel surface is driven by constant changes in saliva composition and swallowing, rendering the benefits of fluoride treatment insufficient [3]. As the excessive use of fluoride is toxic [4], it is important to retain the fluoride applied to the tooth surface for a long time [5]. In recent years, dental coating materials containing bioactive ingredients that suppress tooth sensitivity and that are expected to remineralize teeth have been developed. The uptake of various ions into tooth structures with reduced acid resistance may help to inhibit the progression of dental caries and inhibit biofilm formation.

Thus, we evaluated the antibiofilm properties and demineralization suppression of three dental coating materials with different chemically active components. PRG Barrier Coat^TM^ (PRG) is a tooth-surface coating material that is expected to relieve dentin hypersensitivity. PRG can deposit a 15 μm layer on the surface of teeth. The adhesion strength of PRG to the tooth surface is sufficient although it is not as strong as self-etching or standard phosphoric-acid etching [3]. The filler included in PRG has a high physical strength and can release fluoride (F) and five additional ions (sodium (Na), boron (B), aluminum (Al), silica (Si), and strontium (Sr)) without degrading its material properties [6]. Previous findings demonstrated that the growth of *S. mutans* on the surface was inhibited by resin composites containing fillers at 13.9 (vol. %) or higher. Of the various ions, B and F exhibited the most potent inhibition of *S. mutans* development [7].

BioCoat Ca^TM^ (BioC) is a coating material developed as a hypersensitivity inhibitor and contains 10-methacryloyloxydecyl dihydrogen Ca phosphate (MDCP) and 4-methacryloxyethyl trimellitic acid (C-MET). MDCP is a Ca salt of 10-methacryloyloxydecyl dihydrogen phosphate (MDP), an acidic monomer. MDP electrostatically bonds to the enamel and dentin to produce Ca salts [8]. C-MET is a Ca salt composed of 4-methacryloxyethyl trimellitate (4-MET). 4-MET is a functional acidic monomer that enhances the bonding of the composite resin to the enamel and dentin by chemically binding it to the Ca of residual hydroxyapatite [9]. C-MET has been reported to induce dentin mineralization and promote shear bond strength [10], odontogenic differentiation, and dentine regeneration [11]. A clinical study reported that the combined use of C-MET and MDCP improved cloudiness and brown spots in recently erupted permanent teeth in a clinical study [12].

FluorDental Jelly^TM^ (FluorJ), a fluoride gel (2% sodium fluoride; Bee Brand Medico Dental Co., Ltd., Osaka, Japan) with a fluoride concentration as high as 9000 ppm, was used as the positive control. The antimicrobial mechanisms of fluoride against oral bacteria include increasing the permeability of membranes to protons and limiting the ability of F-ATPases to export protons, which causes cytoplasmic acidification and the acid inhibition of glycolytic enzymes. In addition, fluoride reduces the acid tolerance of bacteria, most effectively at acidic pH values [13,14,15].

These materials may clinically contribute to the suppression of hypersensitivity, remineralization, and caries prevention. However, the mechanism has not been fully elucidated. Thus, this study aimed to investigate the effects of remineralization and *S. mutans* biofilm formation suppression when dental coating materials were applied to early enamel lesions using a modified Robbins device (MRD) flow-cell system.

## 2. Results

### 2.1. Scanning Electron Microscopy (SEM) Observation

**Figure 1:**
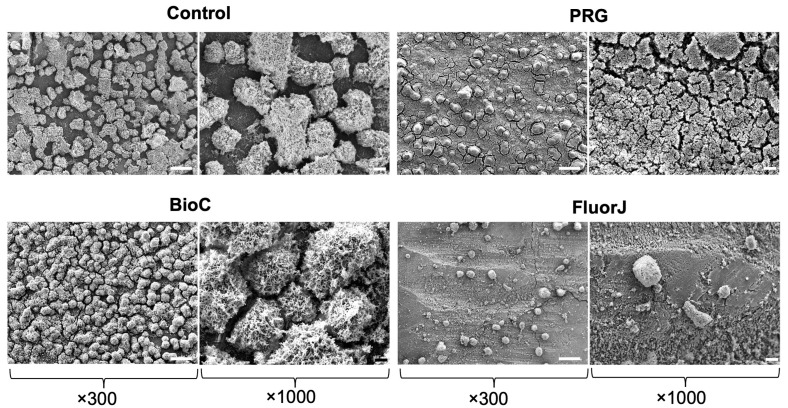
Representative scanning electron microscopy images of *S. mutans* biofilms formed on each specimen after a 24 h incubation. An extremely small number of biofilm clusters were observed on the surface in the FluorJ group. A large number of mature biofilm clusters covered the surface throughout the field of view of the Control, PRG, and BioCoat groups, respectively. Scale bars = 100 μm (×300) and 20 μm (×1000).

Figure 1 shows *S. mutans* on the enamel specimens. *S. mutans* biofilms successfully developed on all materials after a 24 h incubation period. However, biofilm formation in the FluorJ group was remarkably inhibited compared with that in the PRG and BioC groups, exhibiting sparse, thinner biofilm clusters.

### 2.2. Confocal Laser Scanning Microscopy (CLSM) Observation

**Figure 2:**
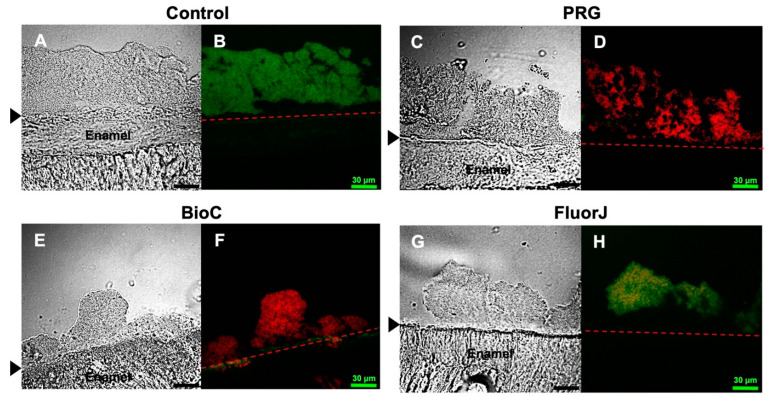
Representative cryosectioned images of *S. mutans* biofilms using Live/Dead^®^ staining. Live cells are stained green (SYTO9) and dead cells are stained red (propidium iodide). (**A**,**C**,**E**,**G**) Transmission images; (**B**,**D**,**F**,**H**) fluorescent images. Black arrow and dash line: interface between enamel surface and biofilm. Scale bar = 30 μm.

The cryosectioned images of *S. mutans* biofilms after 24 h of incubation are shown in Figure 2. The *S. mutans* biofilm was stained with Live/Dead^®^ staining. Live cells were stained green (SYTO9) and dead cells were stained red (propidium iodide). In the control group, most bacteria in the biofilm appeared to be alive with a high biovolume mass. In contrast, the PRG and BioC groups allowed biofilm formation; however, a significant portion of the microorganisms within the biofilm were non-viable. Further, the biovolume in the FluorJ group was notably lower than that in the other groups; however, most bacteria in the FluorJ biofilm remained alive.

### 2.3. Viable and Total Cell Counts of S. mutans Biofilm

**Figure 3:**
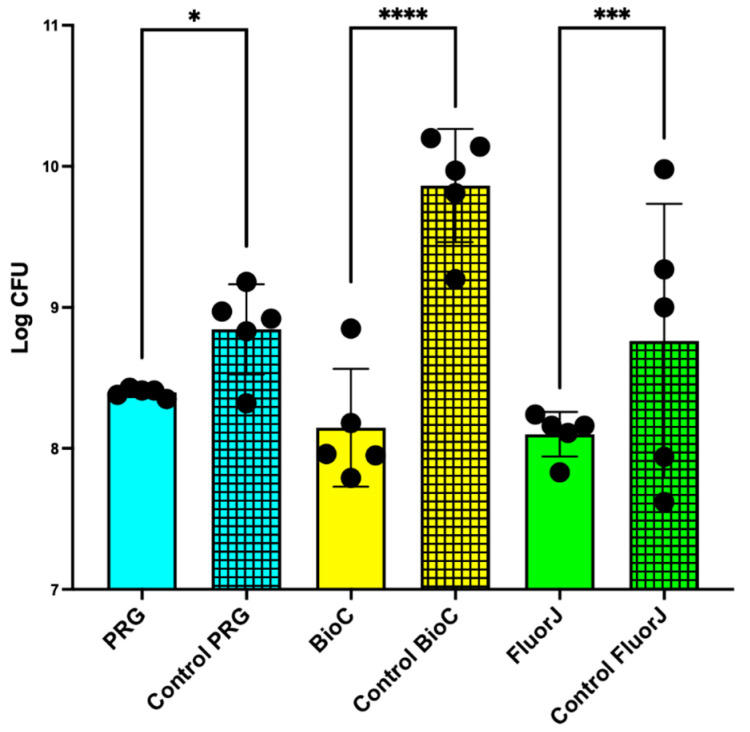
Viable counts of *S. mutans* biofilm cells after incubation for 24 h as determined by colony counts. The results are shown as the means ± the standard deviation of five replicates. The colored grid bars indicate the control group corresponding to each experimental group. **** *p* < 0.0001; *** *p* < 0.001; * *p* < 0.05.

The number of viable cells after 24 h of incubation are shown in Figure 3. A significant difference was observed between the test and corresponding control groups. Notably, BioC exhibited the most significant difference with its control group (*p* < 0.0001) among all tested materials. FluorJ also showed a significant difference from the control group (*p* < 0.001), whereas PRG displayed a comparatively less significant difference than its control group (*p* < 0.05). BioC and FluorJ showed the lowest number of viable cells, which was 1/1000 less than that of the corresponding control group.

**Figure 4:**
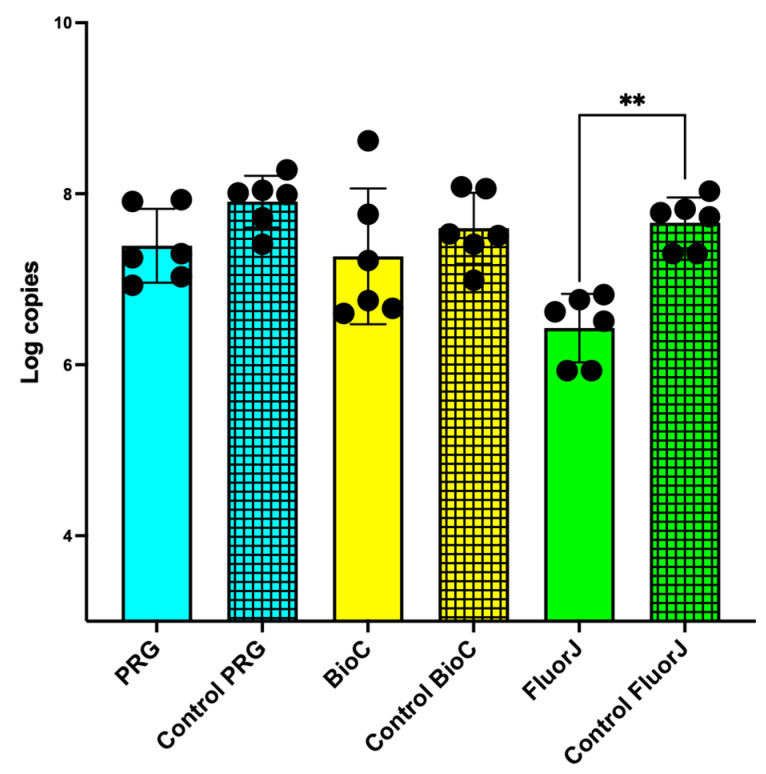
Total bacterial counts of *S. mutans* biofilms formed on PRG, BioC, and FluorJ after a 24 h incubation. The results are shown as the means ± the standard deviations of six replicates. The colored grid bars indicate the control group corresponding to each experimental group. ** *p* < 0.01.

The counts of total bacterial cells in all experimental groups were relatively lower than those in their corresponding control groups; however, FluorJ was the only group showing a significant difference (*p* < 0.01), revealing 6.43 ± 0.40 for the FluorJ group and 7.66 ± 0.30 log copies for the corresponding control (Figure 4). FluorJ was the only material that inhibited biofilm accumulation and had a bactericidal effect.

### 2.4. Adenosine Triphosphate (ATP) Bioluminescence Assay

The number of viable bacteria remaining on the sample surface after detachment was evaluated using an ATP assay (Figure S1). Consistent with the viable cell count (Figure 3), the ATP content in the PRG and BioC experimental groups was significantly lower than that in the control group. FluorJ showed a similar tendency with no significant difference.

### 2.5. Acid Production

**Figure 5:**
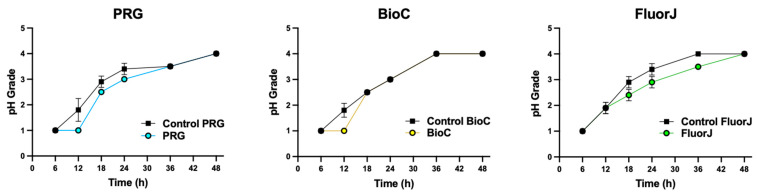
Changes to acid production by biofilm on specimens over 48 h. The pH scoring was ascertained after a predetermined incubation period using a modified 7-scale grading system with a 0.5 interval grade based on a 4-scale system. pH grade 0 had low acidity and grade 5 had high acid-production rates. There was no significant difference between the experimental and the corresponding control group among all test materials.

The acid-producing activity of the biofilms on the specimens was evaluated (Figure 5). Acidification of the media was not significantly affected by the ions contained in the material (*p* > 0.05).

### 2.6. Acid Tolerance

**Figure 6:**
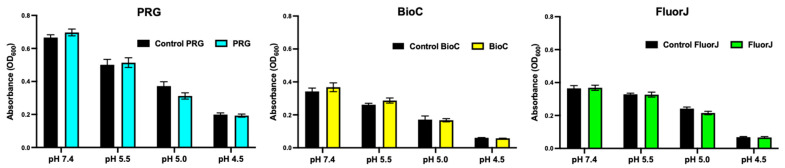
Growth of bacteria in the biofilm formed on the specimens in media with various acidity levels. Bacterial growth was estimated by defining the optical density (OD) value after incubation for 12 h. There was no significant difference between the experimental and the corresponding groups among all test materials.

To analyze the effect of the coating material on the acid tolerance of bacteria in the biofilm, the growth rate of *S. mutans* was evaluated by determining the optical density (OD) after culturing for 12 h in media with various acidity levels (Figure 6). There was no statistically significant difference between the test and corresponding control groups for all test groups (*p* > 0.05).

### 2.7. Gene Expression Analysis

**Figure 7:**
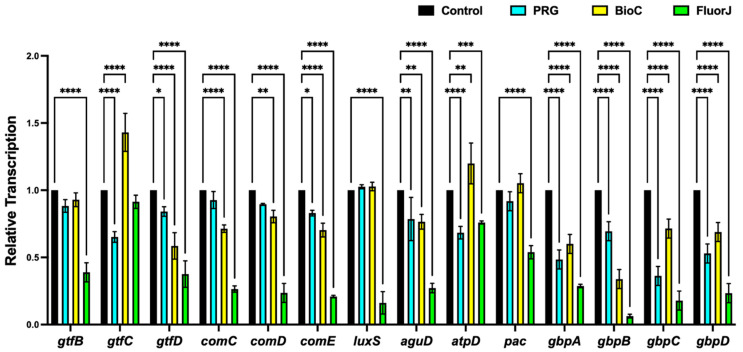
Expression profiles of genes associated with biofilm formation, bacterial adhesion, acid tolerance, and acid production. Results are shown as the mean ± standard deviation of six replicates. **** *p* < 0.0001, *** *p* < 0.001, ** *p* < 0.01, and * *p* < 0.05, as compared with control groups.

The expression profiles of the genes assumed to participate in biofilm formation (*gtfB*, *gtfC*, *gtfD*, *comC*, *comD*, *comE*, and *luxS*), bacterial adherence (*pac*, *gbpA*, *gbpB*, *gbpC*, and *gbpD*), acid tolerance (*aguD*), and acid production (*atpD*) are summarized in Figure 7. *S. mutans* that developed in the FluorJ group showed a significant downregulation in the transcription of all genes compared with the control, except for *gtfC*. The BioC group showed a significant decrease in gene transcription, except for *gtfB*, *gtfC*, *luxS*, *atpD*, and *pac*; however, the decrease was smaller than that in the FluorJ group. The transcription of *gtfC*, *gtfD*, *comE*, *aguD*, *atpD*, and the four gbp genes in the PRG group was significantly downregulated compared with the control group; however, the ratio of downregulation was smaller than that in the FluorJ group.

### 2.8. Electron Probe Microanalyzer (EPMA)

**Figure 8:**
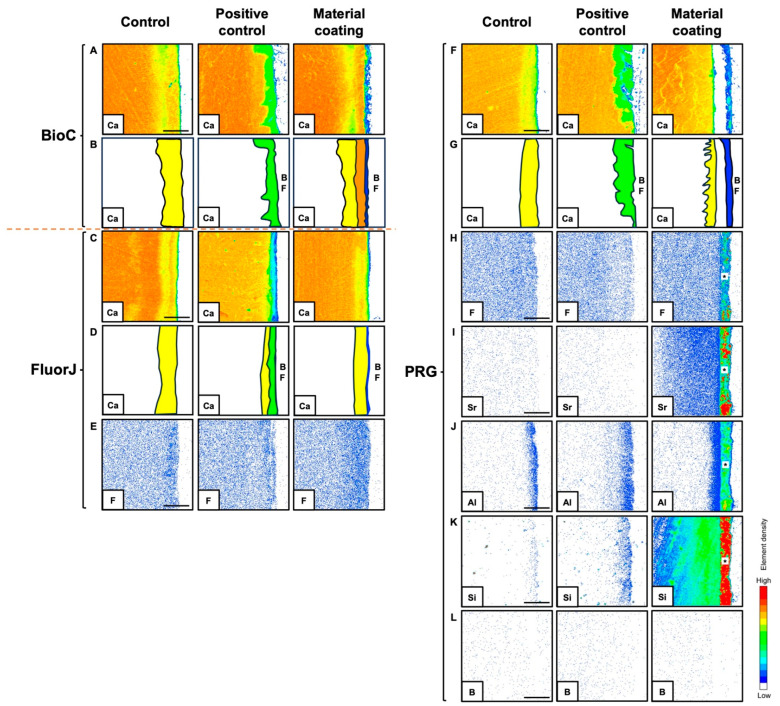
Calcium (Ca), fluorine (F), strontium (Sr), aluminum (Al), silica (Si), and boron (B) ion incorporations into the demineralized bovine enamel after applying BioC (**A**,**B**), FluorJ (**C**–**E**), and PRG (**F**–**L**). Control: no application of coating material. Positive control: biofilm formation without a coating material. Material coating: biofilm formation following application with either coating material. (**B**,**D**,**G**) Illustrations of demineralized areas and Ca ion incorporation; yellow color: experimentally demineralized area using demineralizing solution; green color: demineralized area showing acid production from biofilm; orange color: remineralized area using released Ca ions from coating materials. BF: biofilm. Asterisk (*): PRG. Scale bar = 50 μm.

The elemental mapping images from the EPMA analysis are presented in Figure 8. The analysis revealed ion incorporation into the demineralized layer at varying depths depending on the element. The PRG group showed the incorporation of various ions into the demineralized enamel. The uptake of F and Al ions into the enamel was confined to the superficial surface layer, while B ions did not exhibit ion incorporation. An EPMA probe microanalysis of FluorJ, which contained fluoride ions, showed ion uptake into the demineralized enamel. Enamel demineralization and Ca–ion incorporation were analyzed using Ca mapping (Figure 8A,C,F). Figure 8B,D,G show the demineralized enamel (yellow and green colors in Figure 8B,D,G) and Ca ion uptake (orange color in Figure 8B). The yellow area in the control sample indicated the loss of Ca ions from the demineralizing solution. The green area in the positive control sample further indicated the demineralization of Ca ions due to acid production from the biofilm. All materials showed resistance to acid production from the biofilm. FluorJ (Figure 8C) and PRG (Figure 8F) did not show Ca ion uptake, showing mapping similar to that of the control. BioC showed the incorporation of Ca ions into the enamel underneath the biofilm structure (Figure 8A; material coating).

## 3. Discussion

The primary function of dental coating materials is to serve as a desensitizer. However, because PRG, BioC, and FluorJ contain various active ions and new functional monomers, we expected that they might have antibiofilm effects and might promote remineralization in early enamel lesions. Thus, in this study, we investigated the effects of the tested materials on *S. mutans* biofilms on bovine enamel when these dental coating materials were applied to early enamel lesions by using an MRD device. To overcome the limitations of several other in vitro biofilm model systems, we used an MRD, an in vitro biofilm model system that allows the development of various microbial biofilms under controlled flow conditions [16]. Biofilm formations by oral pathogens have been previously studied using this model system [17,18,19]. The MRD enables simultaneous biofilm formation by various microorganisms on several substrates under various growth conditions. Furthermore, it is simple to transfer discs containing bacterial biofilms from the MRD to another recipient without losing cells. Each type of bovine enamel has a chemical composition similar to that of human enamel [20]. Biofilm maturation and anticariogenic agents can be studied using enamel caries models in humans or bovines [21]. In this study, we used bovine enamel to prepare a pair of standardized specimens from the same tooth, considering the individual differences in its composition.

Regarding antibiofilm effects, FluorJ behaved differently from the other two coating materials. Both the viable and total numbers of bacteria in the FluorJ group significantly decreased compared with those in the control group (Figure 3 and Figure 4). Biofilm formation in the FluorJ group was lower than that in the other groups (Figure 1). These findings suggest that FluorJ inhibited *S. mutans* biofilm formation via bacterial adhesion and antimicrobial activity. The gene expression analysis of the FluorJ group supported this hypothesis. The relative transcription of genes associated with bacterial adhesion (*gbp* genes and *pac*) and biofilm formation (*gtfB*, *gtfD*, *com* genes, and *luxS*) was the lowest for FluorJ among all test materials (Figure 7). Koo et al. reported that fluoride inhibited the production of glucosyltransferases, followed by the suppression of glucan production in *S. mutans*, without interfering with bacterial growth [22]. The inhibition of biofilm formation seems to be associated with starvation because ATP pools and iodophilic polysaccharide levels are remarkably decreased [22]. Fluoride is known to inhibit acidogenicity and acid tolerance [23]. Although the transcription of *aguD* and *atpD* was significantly downregulated (Figure 7), acid production and tolerance were not significantly affected (Figure 5 and Figure 6). These findings indicate that the metabolic alterations caused by fluoride are possibly limited to the bacteria attached to it. This hypothesis can be explained by two factors. One is the restriction of fluoride penetration into biofilms. Watson et al. investigated the penetration of sodium fluoride into a biofilm generated in vivo on a natural enamel surface [24]. The results showed that fluoride penetration increased with exposure time, while the concentration at deeper local sites remained low, indicating the restriction of fluoride penetration. Another possible explanation is the fluid flow in the media. Because released fluoride is washed out by the fluid flow, the uptake of fluoride into bacteria will be limited. The CLSM finding that some bacteria that remained on the FluorJ specimen were viable also supports this hypothesis.

In addition, the antibiofilm effects of FluorJ in clinical practice may be limited to the early stages after its application. It has been reported that fluoride varnish application can affect *S. mutans* biofilm formation; however, antibiofilm activity was reduced over time in an in vitro experiment [25].

The PRG and BioC groups showed a significant reduction in the viable counts of *S. mutans* but no significant difference in total bacterial counts (Figure 3 and Figure 4). Numerous biofilm clusters covered the enamel surface when PRG and BioC were applied (Figure 1), indicating that these two materials failed to inhibit *S. mutans* biofilm formation. These findings suggest that the antibiofilm effects of PRG and BioC were bactericidal. Tanaka et al. tested the antimicrobial activity of PRG on periodontopathogenic biofilms [26]. The results showed that PRG exhibited antibacterial effects against 15 of the 31 pathogens, highlighting its effects on *P. gingivalis*. Yamamoto et al. investigated the effects of PRG on biofilm formation with *S. mutans* using bovine dental specimens and a drip-flow biofilm reactor [27]. The number of bacteria that developed on the PRG-coated specimen, defined as the OD, and that produced insoluble glucan showed a significant decrease compared with the control. Our study supports these results by showing that the transcription of *gtfC* in the PRG group significantly decreased compared with that in the control group.

There are a few reports on the antibiofilm effects of BioC. A previous study reported that biofilm formation was reduced by 65% compared with a control condition after 24 h when *S. mutans* was cultured in a 96-well plate coated with BioC [28]. However, the mechanisms underlying the bactericidal activity of BioC remain unclear. As MDP has no bactericidal effect on *S. mutans* [29], it can be assumed that the bactericidal effect of BioC is due to the acidic property of the adhesive monomer or the supply of excess Ca salts. Ca ions affect various bactericidal and physiological processes, including spore formation, chemotaxis, heterocyst differentiation, transport, and virulence [30]. Elevated levels of free Ca ions can be toxic to bacterial cells and recovery to basal Ca levels is critical for the survival of bacteria and the appropriate regulation of Ca ions [31].

One reason for the biofilm accumulation on the PRG and BioC specimens may have been the roughness of the material surface. The roughness of the restoration surface has been reported to be correlated with biofilm development [32]. Yamamoto et al. reported that the mean surface roughness of bovine dentin treated with PRG was significantly higher than that of the control [27].

Ion incorporation from the tested materials into the enamel was observed using an EPMA. All materials prevented further demineralization from the acid produced by the biofilm (see Figure 8A–G; material coating). It appeared that PRG and BioC mechanically blocked the influence of the acid produced by the biofilm. PRG has been demonstrated to protect exposed roots from both chemical and biological challenges, creating a coating layer with a thickness of approximately 200 μm [6]. Furthermore, dentin coated with PRG has been reported to not cause surface loss underneath *S. mutans* biofilms [27]. During stalling, the uptake of various ions by the demineralized enamel from PRG was achieved, improving the mechanical properties. However, the uptake of F and Al ions was limited to the superficial layer of the enamel surface. BioC recovered the missing Ca ions while preventing acid attacks on the biofilm (Figure 8A; material coating). BioC may contribute to the regeneration of the enamel microstructure by phosphate ions supplied from saliva [10]. FluorJ supplies F ions to demineralized enamel. Fluoride incorporated into the enamel makes the structure less soluble and harder owing to the formation of fluorapatite.

Apart from their effect on early enamel lesions, these coating materials may contribute to protection from other dental diseases. Using a rat pulp injury model, Qiu et al. reported that C-MET produced a continuous hard tissue bridge at the pulp exposure site with favorable biocompatibility [11]. PRG may also be useful in protecting exposed roots, especially at hard-to-access sites [6,33].

Although this study showed that all coating materials could prevent caries progression in vitro, further studies are needed to investigate their actual clinical benefits. Physical barriers can protect the enamel surface from acid attacks through the formation of a hybrid layer; however, some studies reported that coating with adhesives was unable to fully resist acid-attack-induced demineralization [6]. Thus, there may be unexpected interactions among the various ions released from the coating.

Moreover, the optimal approach for the mineralization of early enamel lesions remains unclear. Fluoride remains the gold standard for the arrest of carious lesions as it can drive the incorporation of Ca and P ions into the crystal lattice with the ensuing fluorapatite mineral being significantly more resistant to acid challenges [34]. However, fluoride does not promote the growth of apatite crystals [35]. Thus, the preferred strategy for enamel remineralization has recently shifted from reparative to regenerative approaches (for example, by replacing diseased tooth structures with more biologically similar structures) so that increased Ca and P ions are directly present at the sites to be remineralized [35]. Further well-designed clinical studies are required to clarify whether PRG and BioC offer additional advantages over conventional fluoride applications.

## 4. Materials and Methods

### 4.1. Specimen Preparation

A pair of rectangular-shaped enamel specimens (5 mm × 5 mm × 4 mm) were prepared from 73 bovine upper central incisors using a low-speed diamond saw (Isomet; Buehler, Lake Bluff, IL, USA) under a water coolant. The superficial layer of the enamel was polished using a white stone bur under a water coolant to increase its smoothness and remove stains prior to fabrication. The specimens were immersed in 2.5% sodium hypochlorite (NaOCl) for 1 min and washed with distilled water.

### 4.2. Preparation of Artificial Early Enamel Lesions

To create enamel surface lesions, the entire surface of each specimen, except the enamel surface, was covered with an acid-resistant nail varnish. The specimens were immersed in a demineralizing solution (2.2 mM calcium chloride, 2.2 mM sodium phosphate, and 0.05 M acetic acid with 1 M potassium hydroxide; pH 4.4) for 72 h at 37 °C, along with a stirring rod [36]. The demineralizing solution was changed every 24 h to maintain a constant pH [37].

**Figure 9:**
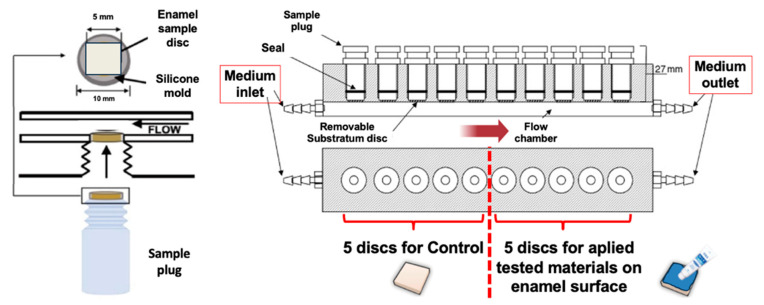
Modified Robbins device (MRD) used in this study. A paired of enamel specimens were mounted on a sampling plug of the MRD. Five plugs served as the experimental group and the other five were allocated as the control group.

The paired enamel specimens were randomly assigned to three material groups: PRG, BioC, and FluorJ (Table 1). One of the paired specimens was allocated to the experimental group and the other served as a control. The samples in the experimental group were treated with either material on the enamel surface according to the manufacturer’s instructions. The specimens were mounted on the sampling plugs of the MRD using a silicone ring (Figure 9). The MRD was sterilized with ethylene oxide gas for 4 h [16].

### 4.3. Bacterial Strains and Culture Conditions

*Streptococcus mutans* strain UA159 was grown overnight from frozen stock in Mitis Salivarius agar (Difco Laboratories, Detroit, MI, USA) at 37 °C under anerobic conditions. The starter cultures were transferred to fresh brain heart infusion (BHI) broth and grown for 6 h at 37 °C under the same conditions. The OD of the bacterial suspension was adjusted to 0.05 at 600 nm prior to inoculation [38].

### 4.4. Adjusted Saliva Preparation and Saliva Pellicle Formation

One of the authors collected the unstimulated saliva samples. Saliva samples were diluted (1:10) with a sterile Ringer solution containing 0.05% cysteine (Sigma-Aldrich, St. Louis, MO, USA). Then, the dilute solution was centrifuged for 10 min at a speed of 10,000× *g* at 4 °C and the supernatant was filter-sterilized [38]. An adjusted saliva solution of 20 mL was pumped into a chamber at a flow rate of 2 mL/min and kept static for 30 min at 37 °C to allow salivary pellicle formation.

### 4.5. Biofilm Formation

Following the salivary pellicle formation, the same volume of *S. mutans* suspension was pumped into the chamber and kept static for 30 min to acquire initial adhesion at 37 °C under anaerobic conditions, as described previously [38]. After 30 min, the medium flow was initiated at a flow rate of 2 mL/min. The medium was 1/10 strength BHI broth supplemented with 0.05% sucrose (pH 7.4) [38]. Bacterial biofilms were anaerobically allowed to develop on the enamel surface for 24 h at 37 °C under the continuous flow condition using the MRD. The flow-cell system consisted of a medium reservoir, peristaltic pump, and carboy for waste, all connected by silicone tubing.

### 4.6. SEM Observation

After incubation, the plug was gently washed twice using sterile phosphate-buffered saline (PBS) (pH 7.0). The specimens were removed from the plug and then fixed with 2.5% glutaraldehyde overnight at 4 °C. Subsequently, the specimens were washed twice with PBS, dehydrated with an ascending series of ethanol (50–100% (*v*/*v*)), dried to a critical point, and subsequently sputtered with gold palladium. The biofilms were observed using SEM at 300× and 1000× magnifications (EPMA-1610; Shimazu, Kyoto, Japan) [39].

### 4.7. Fluorescent Staining and CLSM Observation

The samples were stained using a fluorescent bacterial viability kit (LIVE/DEAD^®^ BacLight^TM^ Bacterial Viability Kit; Thermo Fisher Scientific, Waltham, MA, USA) for 30 min at room temperature in the dark [38,39]. The specimens were embedded in 4% carboxymethyl cellulose sodium salt (Section Lab Co., Ltd., Hiroshima, Japan). The adhesive-film method was used to create a frozen section of 8 µm in thickness vertically along the enamel [40]. Fluorescent tomographic imaging was performed using a CLSM (FV-300; Olympus, Tokyo, Japan) as well as Ar 488 nm and He-Ne 543 nm lasers. Filters of 510–530 nm and ≥610 nm were used to detect SYTO 9 and propidium iodide, respectively [38,39]. A 100× oil immersion objective lens was used. The assay was performed with three replicates per material and a control group.

### 4.8. Viable and Total Cell Counting

After incubation, the plugs were gently washed twice with PBS. The specimens were removed from the plug and transferred to a tube containing 2 mL of PBS. The samples were ultrasonicated for 5 min, vigorously shaken for 1 min, and ultrasonicated again for 5 min. Following biofilm detachment, the suspension was homogenized, serial 10-fold diluted, and plated on Mitis Salivarius agar. Viable cell counting was performed after incubation for 48 h at 37 °C under anaerobic conditions.

For total cell counting, DNA was extracted using a NucleoSpin Microbial DNA kit (Macherey-Nagel, Düren, Germany) according to the manufacturer’s instructions. Total genomic DNA was quantified and stored at 20 °C until further processing. A quantitative analysis of the total bacterial count was performed using a modified Invader PLUS assay (BML, Inc., Tokyo, Japan), as described previously [38].

### 4.9. Adenosine Triphosphate (ATP) Bioluminescence Assay

The number of persistent viable bacteria on the specimens was determined using a BacTiter-Glo^TM^ microbial cell viability assay (Promega Corp., Madison, WI, USA). The luminescence was measured using a GloMax^®^ microplate reader (Promega Corp., Madison, WI, USA) according to a previous study [41].

### 4.10. Acid-Production Testing

Following biofilm detachment after the incubation period, suspensions of *S. mutans* were homogenized, washed, and adjusted to 0.05 for an OD of 600 nm. Approximately 100 µL of the bacterial suspension was added to the test solution of the CAT21 test (caries activity test; Morita Co., Tokyo, Japan) and incubated at 37 °C for 48 h. Acidogenic ability was estimated every 6 h up to 48 h. Scoring was performed using a modified 7-scale grading system with a 0.5 interval grade based on a 4-scale system [42].

### 4.11. Acid-Tolerance Testing

To test acid tolerance, 40 µL of the bacterial suspension, which was prepared in the same manner as in acid-production testing, was added to 160 µL of BHI broths of pH 4.5, 5.0, 5.5, and 7.4 within a Falcon^®^ flat-bottomed polystyrene 96-well plate (Corning Inc. Life Sciences, Durham, NC, USA). After 12 h and 24 h incubation at 37 °C, the OD at 600 nm was measured using a GloMax^®^ microplate reader (Promega corp., Madison, WI, USA) with a slight modification from a previous study [43].

### 4.12. Gene Expression in Relation to Bacterial Adhesion

To investigate the gene transcription associated with biofilm formation, bacterial adherence, acid production, and acid tolerance, *S. mutans* biofilms that had formed on the specimens were collected and washed twice with PBS. The bacterial pellet was resuspended in a TRI reagent (Molecular Research Center, Inc., Cincinnati, OH, USA) and chemically pulverized with Lysing Matrix B using a MagNA Lyser at 7000 rpm for 30 s. RNA isolation was performed using a Direct-zol RNA kit (Zymo Research, Irvine, CA, USA), as described previously [44]. RNA was reverse-transcribed using SuperScript VILO Master Mix (Thermo Fisher Scientific) and qPCR with cDNA was performed using a StepOnePlus real-time PCR system with the SYBR Green detection protocol. The 16S rRNA gene was used as an internal control for data normalization. The primers used in this study are listed in Supplementary Table S1 [45,46,47,48,49,50]. This assay was performed with six replicates per treatment.

### 4.13. EPMA Analysis

After 24 h of incubation, the specimens were embedded in a chemically polymerized resin (Nissin, Kanagawa, Japan). The resin block was cut in half using a diamond disk. The cut surface was serially polished with 2400 grit and 4000 grit SiC paper (Marumoto Struers KK, Tokyo, Japan). Elemental mappings of various ions such as F, Al, Si, Sr, and Ca in early enamel lesions were investigated using a wavelength-dispersive X-ray spectroscopy electron probe microanalyzer with an image observation function (EPMA1601) according to a previous protocol [39,51]. A sample without material or biofilm formation served as the negative control. Biofilms formed without any material served as positive controls.

### 4.14. Statistical Analysis

Statistical analyses were performed using GraphPad Prism 9 software (GraphPad Software, Inc., La Jolla, CA, USA). Where applicable, the data were presented as the mean ± standard deviation. Comparisons between the test material and corresponding control were performed using the Kruskal–Wallis test with a post hoc Steel–Dwass test. Differences were considered to be statistically significant at *p* < 0.05.

## 5. Conclusions

FluorJ exhibited the best antibiofilm effects among the materials used in this study, which may be linked to its antimicrobial properties and inhibition of bacterial adhesion. PRG and BioC failed to prevent bacterial formation; however, these coating materials physically prevented acid attacks from the biofilm while providing ions to the enamel to improve its mechanical properties. Further investigations are required to clarify whether PRG and BioC offer additional advantages over conventional fluoride applications.

## Figures and Tables

**Table 1:** Materials used in this study.

Group	Material	Composition	Application Procedure	Manufacturer
1	PRG Barrier Coat™ (PRG)	Base: S-PRG filler based on fluoro-boroaluminosilicate glass, distilled water, and a methacrylic acid monomer. Active: A phosphonic acid monomer, methacrylic acid monomer Bis-MPEPP, a carboxylic acid monomer, TEGDMA, and a reaction initiator.	(1) Mix one drop of active in the base container. (2) Apply the mixture as thinly as possible to the application area and leave it for at least 3 s. (3) Light cure for 10 s. (4) Gently wipe off the unpolymerized layer on the surface using a cotton ball moistened with water.	Shofu, Kyoto, Japan
2	BioCoat Ca™ (BioC)	Liquid: 4-methacryloxyethyl trimellitic anhydride (4-MET). Brush: Bioactive Monomer™ (10-methacryloyloxydecyl dihydrogen calcium phosphate (MDCP) and 4-methacryloxyethyl trimellitic acid (C-MET)) and a polymerization initiator.	(1) Take one drop of liquid and then mix using a brush for 5 s. (2) Apply the mixture to the tooth surface for 10 s. (3) Blow air gently for 5 s to thinly spread the liquid. (4) Light cure for 5 s. (5) Gently wipe off the unpolymerized layer on the surface using a cotton ball and alcohol.	Sun Medical, Shiga, Japan
3	Fluor Dental Jelly™ (FluorJ)	2% sodium fluoride (NaF) (9000 ppm fluoride), carmellose sodium, and water	Apply onto the surface for 1 min and leave undisturbed for 30 min.	Bee Brand Medico Dental, Osaka, Japan

## Data Availability

The datasets used in the study are available from the corresponding author upon reasonable request.

## Supplementary Materials

The following supporting information can be downloaded at: https://www.mdpi.com/article/10.3390/antibiotics13010106/s1, Figure S1: Relative adenosine triphosphate (ATP) bioluminescence assay content of residual bacterial cells on the sample surface following the biofilm detachment procedure. Table S1: Primer sequences used to analyze the genes associated with biofilm formation, bacterial adhesion, acid production, and tolerance. References [45,46,47,48,49,50] are cited in the supplementary materials.
