# Supplementary material for: Antibiofilm Properties and Demineralization Suppression in Early Enamel Lesions Using Dental Coating Materials
Source: Antibiotics (Basel). 2024 Jan 22;13(1):106. doi: 10.3390/antibiotics13010106 (PMC10812522; doi:10.3390/antibiotics13010106)
Supplement: Supplementary file 1 [file antibiotics-13-00106-s001.zip › antibiotics-2821488-supplementary.pdf]

Table S1 Primer sequences used for analyzing the genes associated with biofilm formation, bacterial adhesion, acid production and acid tolerance.

Gene name	Nucleotide sequence	Reference
16s rRNA	F: 5' -CCATGTGTAGCGGTGAAATGC- 3'	[45]
	R: 5' -TCATCGTTTACGGCGTGGAC- 3'	
gtfB	F: 5' -AGCCGAAAGTTGGTATCGTCC- 3'	[45]
	R: 5' -TGACGCTGTGTTTCTTGGCTC- 3'	
gtfC	F: 5' -TTCCGTCCCTTATTGATGACATG- 3'	[45]
	R: 5' -AATTGAAGCGGACTGGTTGCT- 3'	
gtfD	F: 5' -ACAGCAGACAGCAGCCAAGA- 3'	[45]
	R: 5' -ACTGGGTTTGCTGCGTTTG- 3'	
comD	F: 5' -TTCCTGCAAACTCGATCATATAGG- 3'	[45]
	R: 5' -TGCCAGTTCTGACTTGTTTAGGC- 3'	
comC	F: 5' -GACTAGTCATTGGCGGAAGCCTATCAAC-3	[46]
	R: 5' -GCTCTAGAGCTCAGAACATCAAAAATGACCGTTTAGGAC- 3'	
comE	F: 5' -TTCCTCTGATTGACCATTCTTCTG- 3'	[45]

	R: 5' -GAGTTTATGCCCCTCACTTTTCAG- 3'	
luxS	F: 5' -CCAGGGACATCTTTCCATGAGAT- 3'	[45]
	R: 5' -ACGGGATGATTGACTGTTCCC- 3'	
Pac	F: 5' -AGCTGGAGAGACAAATGGTTCAT- 3'	[47]
	R: 5' -GACACCAGCAGACTTAGCATCTT- 3'	
gbpA	F: 5' -AGGAAACGGCTAAAACCGAA- 3'	[48]
	R: 5' -TTTGATCCCCTGTCTCCACA- 3'	
gbpB	F: 5' -AGTGCTTCGACAGCTGCTGT- 3'	[48]
	R: 5' -AACCGCCATCATTCCATACA- 3'	
gbpC	F: 5' - TCCTCCAGTGACACCACCAA- 3'	[48]
	R: 5' -AAAGTAGCCGCAGCAAATGC- 3'	
gbpD	F: 5' -TTCCGCTTCTAGCCAGCAAT- 3'	[48]
	R: 5' -AATGCGTCGGCTATCGATGT- 3'	
aguD	F: 5' - ATCCCGTGAGTGATAGTATTTG -3'	[49]
	R: 5' -CAAGCCACCAACAAGTAAGG- 3'	

atpD	F: 5' -ACTGGGTTTGCTGCGTTTG- 3'<br>R: 5' -CCAGGCGGTTCATTCATCTGAC- 3'	[50]

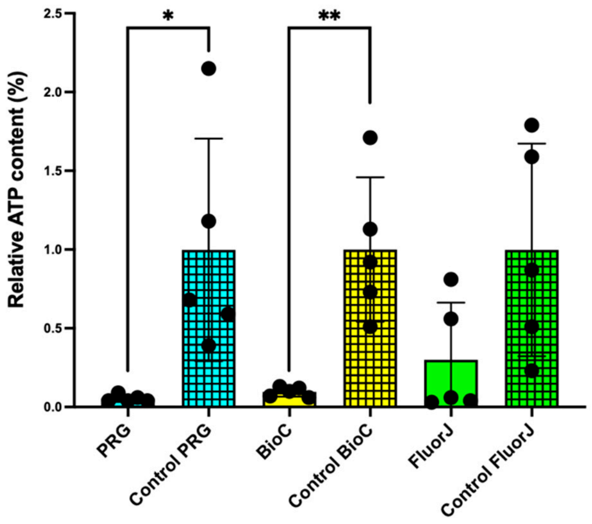

Figure S1 Relative adenosine triphosphate (ATP) bioluminescence assay content of residual bacterial cells on the sample surface following the biofilm detachment procedure. The results are shown as means ± the SD of 5 replicates. **p < 0.01, *p < 0.05.
